# Speculation on the possibility for introducing *Anopheles stephensi* as a species complex: preliminary evidence based on odorant binding protein 1 intron I sequence

**DOI:** 10.1186/s12936-018-2523-y

**Published:** 2018-10-16

**Authors:** Samira Firooziyan, Navid Dinparast Djadid, Saber Gholizadeh

**Affiliations:** 1 Cellular and Molecular Research Center, Urmia University of Medical Sciences, Urmia, P.O. Box: 5756115198, Iran; 2 Medical Entomology Department, School of Public Health, Urmia University of Medical Sciences, Urmia, Iran; 3 Malaria and Vector Research Group, National Insectarium, Pasteur Institute of Iran, Tehran, Iran

**Keywords:** *Anopheles stephensi* complex species, *An. stephensi* sibling A, *An. stephensi* sibling B, *An. stephensi* sibling C

## Abstract

**Background:**

*Anopheles stephensi* is considered an important malaria vector in Iran, Asia, and recently in the Horn of Africa. Recently, *Ansteobp1* intron I sequence has been introduced a new molecular marker for identification of its biological forms including, mysorensis, intermediate and type, using insectary colony specimens.

**Methods:**

In the current study, new marker ability in molecular identification of biological forms has been evaluated with *An. stephensi* specimens collected from Iran and Afghanistan malarious provinces. Following DNA extraction and PCR amplification, sequence analysis and constructed phylogenetic tree revealed that type and intermediate forms are distributed in Iran.

**Results:**

The specimens collected from Afghanistan identified as intermediate and mysorensis forms. Therefore, intermediate form is sympatric species in both countries. Based on the results of *Ansteobp1* intron I sequences, *An. stephensi* could be suggested as new *Anopheles* complex species including *An. stephensi* sibling A (type form), *An. stephensi* sibling B (intermediate form) and *An. stephensi* sibling C (mysorensis form). This is the first report on the presence of *An. stephensi* biological forms in Afghanistan.

**Conclusions:**

Iran is going to eliminate malaria transmission from the country, precise species identification, especially in complex species will be helpful in the prevention of malaria resurgence in the country, mainly because of common fauna of *Anopheles* species and through border malaria and population movement within Afghanistan, Pakistan, and Iran.

## Background

*Anopheles stephensi* is one of the approximately 60 *Anopheles* species considered in malaria transmission as Asian malaria vector [1, 2]. This species is geographically distributed in South Asia and the Arab Peninsula [3, 4]. *Anopheles stephensi* is a key vector in malaria transmission not only, in Iran and India, but also in Asia especially in the Persian Gulf area [1, 3, 5–9]. Recently, this species reported as a malaria vector in the African continent in Djibouti, on the horn of Africa. The high susceptibility of this species to *Plasmodium falciparum* and its tolerance to urban habitats may challenge the global malaria control and elimination programmes in the future [1].

About one century ago, in 1921, malaria was reported from Iran for the first time [10]. Malaria control intervention was started in 1956 in the country [11]. Now, in the Eastern Mediterranean Region, the malaria control programme is in the elimination phase in Iran and Saudi Arabia, however, transmission is still reported from southeastern parts of Iran [12]. The number of malaria cases in Iran has been decreased from 1847 (year) to 81 cases in 2016 [13]. The total number of malaria cases until the end of November 2017 was 800 (736 imported and 63 indigenous) (A. Raeisi, pers. comm.).

So far, there are 30 *Anopheles* species in Iran based on morphological and molecular markers [14], of which four species, including *Anopheles maculipennis*, *Anopheles culicifacies*, *Anopheles fluviatilis,* and *Anopheles superpictus,* belong to species complex based on ribosomal DNA internal transcribed spacer 2 (rDNA-ITS2) sequences [14–20]. However, despite various studies on *An. stephensi*, this species was just considered as races or subspecies [4, 14]. The available information is inadequate to introduce it as a complex species.

A population or series of populations of organisms that are capable of interbreeding freely with each other but not with members of other species is referred to species [21]. There are conflicting reports on crossing experiments between biological or geographical forms of *An. stephensi*. A definite incompatibility existed in cross-mating between *An. stephensi* type and mysorensis biological forms [22]. However, intra-specific variation in the reproductive capacity was demonstrated in this species [23]. On the other hand, any hybrid sterility was not found in the crossing between *An. stephensi* type and mysorensis strains collected from Iran, India, and Iraq [24]. Based on egg morphology characters, *An. stephensi* has three biological forms including type, intermediate and mysorensis. Distribution of all three forms has been reported from malarious regions in Hormozgan, Sistan–Baluchistan, and Fars provinces of Iran [25]. Recently, by using laboratory reared specimens of *An. stephensi*, *AsteObp1* intron I has been introduced as a new molecular marker for the identification of mysorensis, intermediate and type forms of *An*. *stephensi* [4]. In the current study, *AsteObp1* intron I was examined as a molecular marker for identification of *An. stephensi* biological forms on field-collected specimens from the Iran and Afghanistan.

## Methods

### *Anopheles stephensi* mosquitoes

**Fig. 1:**
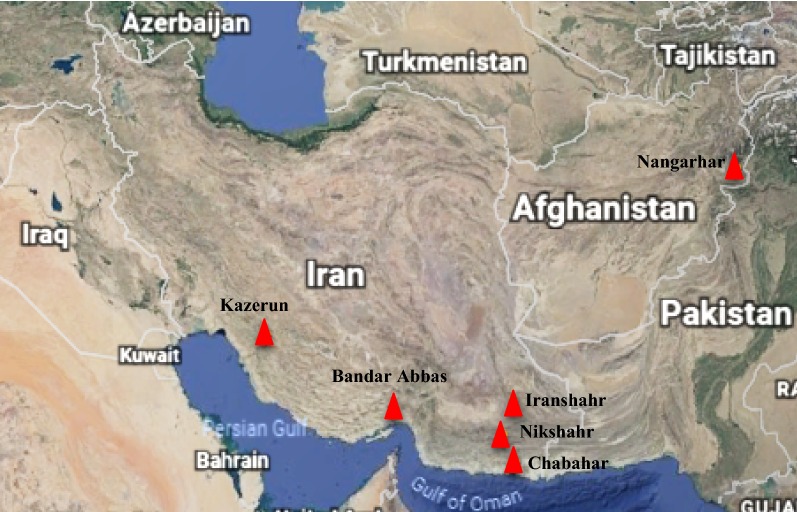
Collection sites of *Anopheles stephensi* specimens in Iran and Afghanistan

Mosquitoes were collected form Hormozgan (Bandar-Abbas district), Sistan and Baluchistan (Chabahar, Nikshahr and Iranshahr districts) and Fars (Kazerun district) provinces in Iran and Nangarhar province (Pol-e-tarache and Ali Khan villages) in Afghanistan by hand catch collection method in 2015 (Fig. 1). The details of sampling locations were presented in Table 1. Adult *An. stephensi* specimens were identified morphologically using keys to the adult females and fourth-instar larvae of the mosquitoes of Iran [26].

**Table 1:** The details of *Anopheles stephensi* samples collected from Iran and Afghanistan

Locations	Latitude (^o^N)	Longitude (^o^E)	Sequenced specimens
Iran			
Bandar Abbas	27°11′11″	56°15′29″	BaU1, BaU3
Chabahar	25°27′39″	60°39′28″	ChU1, ChU2, ChU3, ChU4, ChU5
Nikshahr	26°14′14″	60°13′46″	NiU1
Iranshahr	27°12′22″	60°40′49″	IrU1, IrU3, IrU4
Kazerun	29°37′26″	51°38′57″	KaU3
Afghanistan			
Nangarhar	34°26′03″	70°26′52″	1-H-H, 2-H-H, 3-H-H, 4-H-H, 5-H-H

### DNA extraction and PCR amplification

DNA was extracted from 100 *An. stephensi* specimens using YTA Genomic DNA Extraction Mini Kit (Yekta Tajhiz Azma, Tehran, Iran). Each mosquito was homogenized in the 200-μl TG1 buffer using a micropestle. The mixture was incubated at 60 °C for 1 h, after the addition of 20-μl proteinase K. After incubation, TG2 buffer (200-μl) was added and re-incubated for 10 min at 70 °C. following adding 200-μl cold ethanol, the mixture was transferred to TG mini column and centrifuged for 1 min at 8000 rpm. DNA was washed two times with 500-μl and 700-μl of W1 and wash buffers with a centrifuge for 1 min at 14,000 rpm, respectively. The DNA was eluted from the column using 100-μl elution buffer and stored at − 20 °C until use. The *Ansteobp1* intron I region was amplified using OBP1F1 (CGTAGGTGGAATATAGGTGG) as forward and OBP1R1 (TCGGCGTAACCATATTTGC) as reverse primers [4].

PCR reactions of the *Ansteobp1* intron I region were performed in a 25-μl volume of Master Mix (Yekta Tajhiz Azma, Tehran, Iran). The optimized reactions contained 12.5 μl of Master Mix, 8.5 μl ddH_2_O, 1 μl each of specific primers, and 2 μl of genomic DNA. The amplification profile was set up with a hot start at 95 °C for 5 min, followed by 30 cycles of denaturation at 95 °C for 1 min, annealing at 60 °C for 1.20 min, and extension at 72 °C for 1.20 min with an additional 10 min extension time in the last cycle. PCR products were visualized on a 0.8% agarose gel containing safe stain and using a UV transilluminator. The sequencing of amplified fragment in representative samples was performed in an ABI377 automatic sequencer by using the same both amplification primers.

### Sequence analysis

The intron I sequence on *An. stephensi* Odorant Binding Protein 1 (*Ansteobp1*) gene was analysed using the Basic Local Alignment Search Tool (BLAST) (http://www.ncbi.nlm.nih.gov/blast/) and double checked with Chromas software version 2.31 (http://www.technelysium.com.au/chromas.html). The sequences related to different forms of *An. stephensi* were aligned and compared using Clustal Omega [27]. The final sequences were aligned with three representative sequences in the GenBank. The phylogenetic tree was constructed using distance Neighbor-joining and maximum likelihood Methods based on the Tamura–Nei model’s model in Molecular Evolutionary Genetics Analysis version 6.0. (MEGA6) [28]. Nucleotide sequences are available in the GenBank, European Molecular Biology Laboratory (EMBL), and DNA Data Bank of Japan (DDBJ) databases [GenBank ID: MG797525-MG797537].

## Results

An 845 bp fragment was amplified in 100 field-collected specimens from Iran and Afghanistan using OBP1F1 and OBP1R1 primers [4]. In total, 18 specimens were applied for direct sequencing form Iran (n = 13) and Afghanistan (n = 5), randomly. The length of intron I region was 115 bp and 120 bp of sequenced specimens. The comparison of these sequences with representative mysorensis [GenBank: KJ557449], intermediate (KJ557452) and type (KJ557463) biological forms intron I sequences showed that Afghani specimens were mysorensis (n = 2) and type (n = 3) forms, while Iranian specimens were intermediate (n = 4) and type (n = 9) forms. Therefore, *An. stephensi* type form was the prevalent biological form in Iranian specimens.

**Fig. 2:**
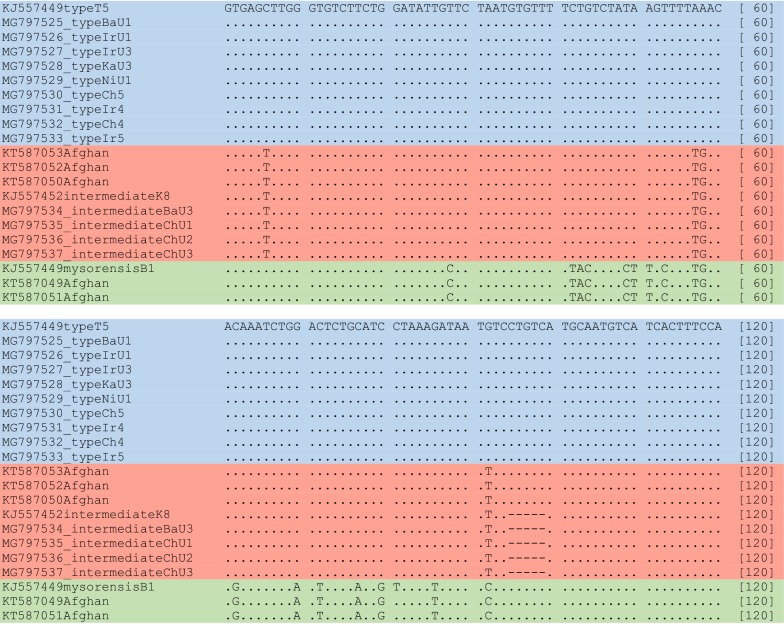
Multiple sequence alignment of Obp1 intron I region of 21 specimens of field-collected specimens of *Anopheles stephensi* biological forms. Sequences with accession numbers KJ557463 (T5), BaU1, IrU1, IrU3, KaU3, NiU1, Ch5, Ir4, Ch4, Ir5, KT587050, KT587052, and KT587053 are related to *An. stephensi* type (Blue), KJ557452 (K8), BaU3, ChU1, ChU2 and ChU3 to *An. stephensi* intermediate (Red), and KJ557449 (B1), KT587049 and KT587051 to *An. stephensi* mysorensis (Green) forms. A dot indicates identity with the reference sequence and a dash indicates a deletion

**Fig. 3:**
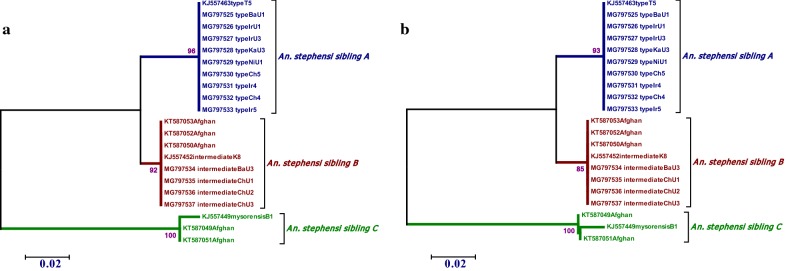
Maximum likelihood (**a**) and neighbor-joining (**b**) phylogenetic trees based on AnsteObp1 intron I fragments for *Anopheles stephensi* specimens collected from Iran and Afghanistan. The bootstrap consensus tree inferred from 1000 replicates is taken to represent the evolutionary history of the taxa analyzed. Bootstrap values > 50% has been shown above each node. All positions containing gaps and missing data were eliminated from the dataset (complete deletion option). GenBank ID: KJ557449, KJ557452, and KJ557463 were used as representative sequences for *An. stephensi* mysorensis, intermediate and type biological forms Obp1 intron I sequence [4]

The sequences obtained from *An. stephensi* specimens collected from Afghanistan were two groups. The sequence similarity within each group was 100%, while it was 86.67% between both groups. The multiple sequence alignment of KT587049 and KT587051 with representative mysorensis, intermediate and type biological forms showed 99.17% similarity with mysorensis (KJ557449). A 0.83% sequence variation was because of a nucleotide transition/transversion (T/C) in position 81 (Fig. 2). The second group of Afghani sequences, KT587050, KT587052, and KT587053, were a combination of type and intermediate forms. They were 96.67% similar to representative type form, and 90.43% to intermediate because of five nucleotide insertion in position 94–99 (Fig. 2). Interestingly, when the phylogenetic tree was constructed, they were placed with intermediate sequences in the same branch (Fig. 3).

The comparison of Iranian field collected *An. stephensi Asteobp1* intron I sequence with representative biological forms sequence categorized them into intermediate (BaU3, ChU1, ChU2, and ChU3) and type (BaU1, IrU1, IrU3, KaU3, NiU1, Ch5, Ir4, Ch4, and Ir5) with 100% similarity. The nucleotide sequence variation between these two forms was 13.04%.

The sequence similarity in *Ansteobp1* intron I region within field-collected specimens of *An. stephensi* biological forms were 100% but it was 75.65–86.96% between biological forms. Therefore, neighbor-joining and maximum likelihood phylogenetic trees, constructed based on *Ansteobp1* intron I sequence demonstrated a similar topology in type and intermediate branches, while, mysorensis branch was varied in both phylogenetic trees (Fig. 3). Phylogenetic tree constructed based on the maximum-likelihood algorithm in the current study had close proximity with trees constructed in our recent study based on insectary-reared specimens [4].

## Discussion

Among three biological forms of *An. stephensi*, the majority of malaria transmission was carried out by type form in its range [3]. Recently, the genome of the Indian strain of *An. stephensi* was analysed [2]. In addition, the distribution and possible role of this major urban malaria vector in the resurgence of malaria in Africa have been reported [1]. The form of collected *An. stephensi* strain from the horn of Africa is not clear, however, it could be suggested that they can use Ansteobp1 intron I sequence to determine it, which will be helpful in rapid interruption of malaria transmission cycle in the region and complete eradication of *An. stephensi* from African countries.

All of three biological forms of *An. stephensi* have been distributed in malarious provinces of Iran including Sistan and Baluchistan, Hormozgan and Fars [25]. In the current study, *An. stephensi* specimens collected from Iranshahr (Sistan and Baluchistan province) and Kazerun (Fars province) were determined as type (*An. stephensi* sibling A), while, Bandar-Abbas (Hormozgan province) and Chabahar (Sistan and Baluchistan province) specimens were both type (*An. stephensi* sibling A) and intermediate (*An. stephensi* sibling B) forms. The intermediate (*An. stephensi* sibling B) and mysorensis (*An. stephensi* sibling C) were the forms collected from Nangarhar province in Afghanistan. Therefore, the distribution of intermediate form (*An. stephensi* sibling B) was reported in both countries, Iran and Afghanistan.

*Anopheles stephensi* was classified into two variety based on the number of egg ridges in 1937 [29]. Since these numbers have extensive overlap in intermediate form, it may not be possible to detect forms accurately; therefore, the cross may occur between the same biological forms. This hypothesis may be the reason for conflicting results in crossing experiments. Therefore, it is recommended that cross-experiments be performed after accurate identification of the forms with molecular markers, such as obp1 intron I. Earlier, various studies used chromosomal karyotypes in differentiation between the rural and urban population of *An. stephensi* [30–32]. They reported that rural and urban populations of this species are different races.

The precise identification of *Anopheles* species is very important in malaria surveillance, control, and elimination programs. Mitochondrial and DNA-based methods were used to identification of *An. stephensi*, *Anopheles culicifacies, Anopheles superpictus, Anopheles maculipennis, Anopheles fluviatilis, Anopheles sacharovi, Anopheles dthali,* and *Anopheles pulcherrimus* species reported as malaria vectors in Iran, based on rDNA-ITS2 sequence, however, *An. maculipennis, An. culicifacies* and *An. fluviatilis* belong to complex species [15–17, 33, 34]. Recently, *An. superpictus* introduced a suspected cryptic species complex, based on molecular phylogenetic analysis of Iranian anophelines [14, 20]. In addition, *An. stephensi* still under genetic dissociation within its different biological forms including, type, Intermediate and mysorensis, and is considered as a suspected cryptic species complex. ITS2 and D3 loci showed identical nucleotide sequences in type and mysorensis biological forms suggested that these molecular markers are not suitable for the identification of biological forms [35]. On the other hand, *Anopheles persiensis* was characterized and named principally as a new record to the world and Iranian *Anopheles* fauna based on DNA evidence for the first time [36] without any crossing experiment between *An. maculipennis* complex members. These markers seem not to be the proper markers for isolating and identifying the biological forms of this species. However, it was shown that three biological forms of *An. stephensi* insectary strains could be easily determined using *Ansteobp1* intron I sequence [4]. In the current study, the idea was examined using field-collected *An. stephensi* specimens from Sistan and Baluchistan, Hormozgan and Fars provinces in Iran and Nangarhar province in Afghanistan. Sequence analysis confirms that *Ansteobp1* intron I sequence could be introduced as a molecular marker for the detection of three biological forms (Fig. 3). Therefore, *An. stephensi* could be considered as complex species including *An. stephensi* sibling A (type), *An. stephensi* sibling B (intermediate) and *An. stephensi* sibling C (mysorensis).

## Conclusions

The current study reports mysorensis and intermediated biological forms of *An. stephensi* in Afghanistan for the first time. The distribution pattern of the biological form using a new molecular marker with field specimens is not compatible with their distribution based on egg morphology. It seems that *An. stephensi* could be assumed as new malaria vector complex species and should be considered in malaria control and elimination programmes.

## Abbreviations

rDNA-ITS2: ribosomal DNA internal transcribed spacer 2
*Ansteobp1*: *An. stephensi* odorant binding protein 1
BLAST: Basic Local Alignment Search Tool
MEGA6: Molecular Evolutionary Genetics Analysis version 6.0
EMBL: European Molecular Biology Laboratory
DDBJ: DNA Data Bank of Japan
